# Physicochemical Characteristics of Chitosan-Based Hydrogels Containing Albumin Particles and *Aloe vera* Juice as Transdermal Systems Functionalized in the Viewpoint of Potential Biomedical Applications

**DOI:** 10.3390/ma14195832

**Published:** 2021-10-06

**Authors:** Sonia Kudłacik-Kramarczyk, Magdalena Głąb, Anna Drabczyk, Aleksandra Kordyka, Marcin Godzierz, Paweł S. Wróbel, Marcel Krzan, Marimuthu Uthayakumar, Magdalena Kędzierska, Bożena Tyliszczak

**Affiliations:** 1 Department of Materials Science, Faculty of Materials Engineering and Physics, Cracow University of Technology, 37 Jana Pawła II Av., 31-864 Krakow, Poland; bozena.tyliszczak@pk.edu.pl; 2 Centre of Polymer and Carbon Materials, Polish Academy of Sciences; 34 M. Curie-Skłodowskiej St., 41-819 Zabrze, Poland; akordyka@cmpw-pan.edu.pl (A.K.); mgodzierz@cmpw-pan.edu.pl (M.G.); pwrobel@cmpw-pan.edu.pl (P.S.W.); 3 Jerzy Haber Institute of Catalysis and Surface Chemistry, Polish Academy of Sciences, 8 Niezapominajek St., 30-239 Krakow, Poland; marcel.krzan@ikifp.edu.pl; 4 Faculty of Mechanical Engineering, Kalasalingam Academy of Research and Education, Krishnankoil 626126, India; uthaykumar@gmail.com; 5 Department of Chemotherapy, Medical University of Lodz, WWCOiT Copernicus Hospital, 90-001 Lodz, Poland; kameleonmagda6@gmail.com

**Keywords:** chitosan hydrogels, albumin particles, *Aloe vera* juice, wettability, tensile load, swelling ability, hydrogel dressings

## Abstract

In recent years, many investigations on the development of innovative dressing materials with potential applications, e.g., for cytostatics delivery, have been performed. One of the most promising carriers is albumin, which tends to accumulate near cancer cells. Here, chitosan-based hydrogels containing albumin spheres and *Aloe vera* juice, designed for the treatment of skin cancers or burn wounds resulting from radiotherapy, were developed. The presence of albumin in hydrogel matrices was confirmed via Fourier transform infrared (FT-IR) and Raman spectroscopy. Albumin spheres were clearly visible in microscopic images. It was proved that the introduction of albumin into hydrogels resulted in their increased resistance to the tensile load, i.e., approximately 30% more force was needed to break such materials. Modified hydrogels showed approximately 10% more swelling ability. All hydrogels were characterized by hydrophilicity (contact angles were <90°) which may support the regeneration of epithelial cells and non-cytotoxicity towards murine fibroblasts L929 and released *Aloe vera* juice more effectively in an acidic environment than in a neutral one wherein spheres introduced into the hydrogel matrix extended the release time. Thus, the developed materials, due to their chemical composition and physicochemical properties, constitute promising materials with great application potential for biomedical purposes.

## 1. Introduction

A transdermal plaster is a self-adhesive dressing material whose main task is to non-invasively deliver a specific dose of the active substance into the bloodstream, wherein the drug is transported through the skin in a continuous or controlled manner [1]. In addition to controlled drug release, the application of such transdermal systems may increase patient comfort compared to other systems in which the drug is delivered intravenously or intramuscularly. Furthermore, transdermal drug delivery limits the possibility of the gastric degradation of substances occurring in the case of oral drug delivery systems [2,3]. The main issue of the application of transdermal systems is the fact that the skin constitutes an effective protective barrier. Thus, only selected therapeutics show the ability to penetrate it [4]. Nonetheless, the administration of therapeutics via the transdermal route was already described in ancient medical records. For example, in Ancient Egypt, the application of various types of ointments as well as plasters based on natural (plant or animal origin) extracts was widely known [5,6]. The main development of transdermal plasters took place in the second half of the 20th century. Currently, many investigations on the development of the methodology of drug delivery through the skin in a precise and repeatable manner leading to the desired systemic effects are being performed [7,8].

Sarheed et al. conducted studies on the delivery of lidocaine, i.e., an active substance used as a local anesthetic. The oil-in-water nanoemulsions were proposed as drug carriers because such systems are considered particularly suitable for encapsulation of lipophilic molecules. The prepared drug delivery systems showed stability and allowed the release of the drug through the skin within 24 and 48 h [9]. Transdermal plasters also based on the emulsions have been developed, e.g., by Patel et al. [10] and Fardous et al. [11]. Next, Rasekh et al. proposed transdermal systems based on polyvinylpyrrolidone (PVP) fibers containing a non-steroidal anti-inflammatory drug (indomethacin). Electrospinning was chosen for the preparation of such modified PVP fibers. The encapsulation efficiency was 75% while the complete drug release was achieved within 45 min [12]. In turn, the main purpose of the investigations performed by Idres et al. was to determine the impact of various additives on the release of flurbiprofen from transdermal plasters. Modifiers such as poly(ethylene glycol), dibutyl phthalate, Span 20, sodium lauryl sulfate, isopropyl myristate, and ethanol were analyzed. The main component of the transdermal systems tested was ethyl cellulose. It was proved that the most favorable properties related to the release of the drug showed ethyl cellulose-based transdermal systems modified with isopropyl myristate and poly(ethylene glycol) [13]. An interesting solution applied in the transdermal drug delivery systems are SmartFilms materials. The method based on these systems was developed mainly for the delivery of drugs which are poorly soluble in water. Such a delivery involves the formation of a thin film containing an active substance which is a three-step process. Firstly, a drug is dissolved in a suitable solvent. Next, the solution obtained is applied to a cellulose matrix (e.g., a paper). The final step involves the solvent evaporation. Such a procedure is repeated several times until the desired drug concentration is achieved. Then, the films obtained are ready to be used as transdermal plasters [14].

Among the applied transdermal plasters, particular attention is paid to the hydrogels. Various systems based on these polymers are being investigated as transdermal drug delivery systems [15,16]. For example, Jung et al. performed studies on hydrogels based on polyacrylamide/polydopamine embedded in mesoporous silica nanoparticles. The mentioned nanoparticles with very large pores were used to improve the cohesive properties between them and polymer chains. Moreover, the proposed system showed a high strength and a good adhesion to the skin [17]. Next, Viyoch et al. developed a transdermal hydrogel plaster using a starch-crosslinked chitosan as a polymer matrix. The substance which was released from such a matrix was analyzed as α-hydroxy acid contained in the tamarind pulp extract. [18]. Hydrogels based on the same reagent and containing curcuminoids applied for cosmetic purposes were presented in [19]. In turn, Ishii et al. proposed hydrogel plasters as materials with application potential for non-invasive transdermal vaccination [20]. Another interesting study whose main purpose was to develop hydrogels containing vinorelbine (a cytostatic drug widely used in melanoma treatment) was performed by Fonceca et al. [21].

The interest in transdermal plasters is constantly growing. Nonetheless, only a few literature reports have focused on the delivery of the cytostatic drugs used in cancer treatment via transdermal systems. Here, investigations are presented whose main purpose was to obtain and characterize the hydrogel materials with potential application for biomedical purposes in melanoma treatment or in the treatment of burn wounds caused by radiotherapy. As a main component of the developed hydrogel matrices, chitosan was selected. Additionally, obtained materials were modified with *Aloe vera* extract and albumin particles. *Aloe vera* was used due to its well-known anti-inflammatory and antibacterial properties, which may support the wound healing process [22,23]. In turn, albumin tends to agglomerate near inflamed and neoplastic tissues, which makes it an excellent carrier for anticancer drugs [24,25]. In this paper, the synthesis of hydrogel polymers modified both with albumin spheres and *Aloe vera* juice is presented. The functional groups and chemical bonds present in the structures of the prepared materials were verified via spectroscopic methods including Raman spectroscopy and Fourier transform infrared (FT-IR) spectroscopy. Next, prepared hydrogels were subjected to the tensile load while the changes in their crystallinity associated with this tension were evaluated. The surface morphology of the hydrogels was characterized via scanning electron microscopy (SEM). The wettability and the swelling properties of the obtained hydrogels were also verified while their sorption properties were particularly important in the viewpoint of potential absorption of wound exudate by hydrogel dressings. Additionally, the release profile of *Aloe vera* from developed polymers was investigated. An important aspect of the performed experiment was the biological assessment of the hydrogels, which was conducted via MTT reduction assay and using murine fibroblasts L929.

## 2. Materials and Methods

### 2.1. Materials

Chitosan (high molecular weight; deacetylation degree 75–85%), diacrylate poly(ethylene glycol) (PEGDA; crosslinking agent; average molecular weight M_n_ = 700 g/mol), and 2-hydroxy-2-methylpropiophenone (photoinitiator; 97%; d = 1.077 g/mL) were purchased from Sigma Aldrich (Saint Louis, MO, USA). *Aloe vera* juice (99.5%) was purchased from Herbal Pharmaceuticals (Kraków, Poland). Albumin (from chicken egg whites, lyophilized powder) and hydrochloric acid (35–38%; d = 1.19 g/mL) were purchased from Avantor Performance Materials Poland S.A. (Gliwice, Poland). Next, potassium phosphate, phosphate buffered saline (PBS, tablets) and tris(hydroxymethyl)aminomethane (≥99.8%, ACS reagent) were purchased from Merck (Darmstadt, Germany). Acetate buffer solution (pH = 5.5) was purchased from Pol-Aura (Zabrze, Poland).

### 2.2. The Synthesis Methodology of the Hydrogels

The synthesis methodology of the hydrogels as well as the selection of the amount of the individual reagents included in the final materials were presented in detail in our previous works [26,27]. In brief, to start, 1.0% chitosan solution in 0.05% acetic acid solution (the base solution) was prepared. Next, the adequate amount of *Aloe vera* juice was introduced into the solution. After mixing the mixture obtained, the adequate amounts of the crosslinking agent (PEGDA) and the photoinitiator (2-hydroxy-2-methylpropiophenone) were added. The whole mixture was mixed intensively, poured into a Petri dish, and treated with UV radiation for 120 s. The photopolymerization process was conducted using an EMITA VP-60 lamp (power: 180 W, λ = 320 nm). The amounts of the reagents applied for the preparation of the hydrogels modified with *Aloe vera* juice are presented in Table 1.

Next, to prepare the albumin nanoparticles for use in further syntheses as the modifying agent of the hydrogels, the salt-induced precipitation process was selected. Albumin was firstly dissolved in a Tris-HCl solution (pH = 7); two solutions were prepared in this way, with concentrations of 4.0 mg/mL and 8.0 mg/mL, respectively. Then, the albumin solutions were mixed with a salting-out agent (i.e., 2 M solution of potassium phosphate) in a volume ratio of 1:1. The precipitated albumin nanoparticles were subsequently centrifuged at room temperature for 15 min (80 rpm) and suspended in a PBS solution. The synthesis methodology of albumin spheres as well as their purification were accurately described in [28]. The hydrogel polymers containing albumin nanoparticles were prepared in the same manner as the unmodified hydrogels, i.e., as was described in the previous paragraph. Apart from all the reagents, adequate amounts of albumin nanoparticles in the PBS solution were introduced into the reaction mixture and the whole mixture was—as before—treated with UV radiation. Detailed amounts of the reagents used for the preparation of both the hydrogels with and without albumin nanoparticles are indicated in Table 1.

**Figure 1:**
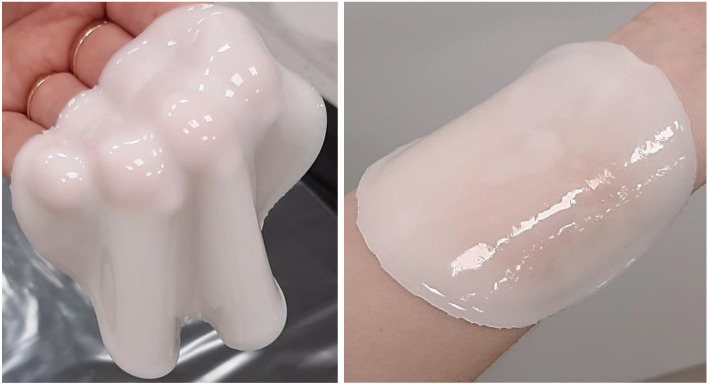
Example images of obtained hydrogels.

In Figure 1 below, example images of the obtained hydrogel polymers are presented.

The obtained hydrogels were subsequently subjected to numerous studies to determine their physicochemical properties.

### 2.3. Methodology of the Investigations

#### 2.3.1. Structural Analysis of the Hydrogels via Fourier Transform Infrared (FT-IR) Spectroscopy

In order to verify the functional groups present in the structure of the obtained polymers, FT-IR spectroscopy was performed. The measurements of all the hydrogels were conducted at room temperature using a Spectrum 65 (Perkin Elmer, Waltham, MA, USA) spectrometer equipped with an attenuated total reflection (ATR) attachment containing a diamond/ZnSe crystal. The measurement range was 4000–600 cm^−1^ (32 scans at 4.0 cm^−1^ resolution).

#### 2.3.2. Evaluation of the Hydrogels via Raman Spectroscopy

The study was conducted using a Witec Alpha M300+ (WITec Corp., Ulm, Germany) Raman spectrometer with a confocal microscope and a Nd-YAG laser operated with an excitation wavelength of 532 nm and power of 50 mW. The main purpose of the research was to analyze the surfaces of the hydrogels. The spectrometer was calibrated to a 520 cm^−1^ Si signal. The measurements were conducted with an integration time of 2 s and 300 accumulations.

#### 2.3.3. Analysis of the Crystallinity of the Hydrogels under the Tensile Load via the X-ray Diffraction (XRD) Technique

X-ray analysis of chitosan-based hydrogels during tensile load was performed using a DEBEN microtensile stage mounted on a D8 Advance diffractometer (Bruker, Karlsruhe, Germany) with a Cu-Kα cathode (λ = 1.54 Å). The diffractometer was operating at 40 kV voltage, 40 mA current, and with a LYNXEYE XE-T detector. The scan rate was 4.8°/min with a scanning step of 0.02° in the range of 5°–70° 2Θ. In tensile tests, a 2 kN crosshead was used, with a stretching speed of 0.4 mm/min. Each specimen had a 10 mm^2^ cross-section and was 14.5 mm long and they were extended by 4 mm.

#### 2.3.4. Morphological Analysis of the Hydrogels via Scanning Electron Microscopy (SEM)

Scanning electron microscopy was performed to characterize the surface morphology of the obtained hydrogels. For this purpose, a scanning electron microscope Jeol 5510LV (Akishima, Tokyo, Japan) equipped with an EDS (Energy Dispersive X-ray) detector with an IXRF (X-ray source) system (Freising, Germany) was used. Before the measurements, the analyzed hydrogel samples were dried and subjected to gold sputtering (to improve their conductivity). Analyses were conducted to verify the impact of the albumin nanoparticles on the surface morphology of the hydrogels.

#### 2.3.5. Studies on the Swelling Properties of the Hydrogels

The next study was performed to characterize the sorption properties of both the unmodified hydrogels and the hydrogels containing albumin. For this purpose, hydrogel samples weighing approximately 1.0 g were cut from the obtained materials and introduced into 50 mL of the selected liquids. After selected periods of time—i.e., 1 h, 24 h, 48 h, and 72 h—the samples were separated from the tested liquids, excess water was removed using a paper towel, and the samples were weighed again. The swelling ability of the hydrogels was defined using the swelling ratio (α), which was calculated by means of the Equation (1):

(1) $$\alpha =\frac{\left(m-m_{o}\right)}{m_{o}}$$

where α is the swelling ratio, g/g; m is the mass of swollen hydrogel, g; and m_o_ is the mass of dry hydrogel, g.

Analyses were conducted using such liquids as distilled water, Ringer liquid (fluid isotonic to human blood), and simulated body fluid (SBF, isotonic to human blood plasma). The concentrations of ions present in the SBF solution are presented in Table 2.

#### 2.3.6. Wettability Measurements of the Hydrogels

The subsequent study was focused on determining the wetting properties of the hydrogels. These were characterized by defining the dynamic contact angle between the tested hydrogel sample and the liquid. Such analyses were performed using the Drop Shape Analyzer Kruss DSA100M optical contact angle measuring instrument (Gmbh, Hamburg, Germany). Sterile syringe needles (NE 44, Kruss Gmbh, Hamburg, Germany) were used for each new analysis. The Kruss DSA100M uses an optical microscope and a digital camera (200 fps) which quickly takes images of the tested material and uses a digital image processing algorithm to calculate the contact angle of the droplet based on the Laplace–Young approximations or tangents. The device selected for measurements is characterized by a high precision, accurate measurement results, simple operation, and good reproducibility. The accuracy of the instrument is improved by using a particular environmental cell. During the measurements, the test chamber temperature was controlled using a thermostatic water bath, which allowed a constant humidity and constant temperature conditions (22.0 ± 0.3 °C) to be maintained. For each tested sample, more than three successive analyses were performed.

To perform an analysis, the hydrogel samples were inputted into the test platform inside the environmental cell with a fixed and installed syringe and then the position of the needle was adjusted. The contact angle was recorded on the video and measured after the determination of the baseline. Once the liquid dropped onto the sample reached equilibrium, the instrument showed the calculated contact angle value. The same methodology was applied for each used solvent (i.e., water δ = 72.30 mN/m, diiodomethane δ = 50.80 mN/m). In the experiments, pure double distilled water (Millipore Q, 18.60 mΩ/cm) was used. Before the measurements, surface tension analyses aimed at determining the purity of the water were carried out. The equilibrium surface tension (δ = 72.30 mN/m) of the water was verified using the Drop Shape Analyser DSA100 (Kruss, Gmbh) according to the pendant drop method performed by means of the sterile syringe needles (NE43, Kruss).

Furthermore, the surface free energy was also investigated for a more accurate analysis of wettability. For determining this parameter, the Owens–Wendt method [30], which is generally accepted as the best for polymer substances evaluations, was applied.

In this analysis, two liquids, i.e., bipolar water and polar diiodomethane, were used to characterize the surface free energy of the examined hydrogels. For this purpose, the Owens–Wendt method was used, which assumes that interactions between molecules of two substances present in their surface layer are equal to the geometric average of the intermolecular interactions within each substance. A detailed introduction to this method was presented by Rudawska et al. [31]. According to the assumptions of this method, the polar and dispersive components of the tested materials can be calculated using Equations (2) and (3) [31]:

(2) $${\left(\gamma_{S}^{D}\right)}^{0.5}=\;\frac{\gamma_{d}\left(cos\theta_{d\;}+1\right)-\sqrt{\frac{\gamma_{d}^{P}}{\gamma_{w}^{P}}\gamma_{w}\left(cos\theta_{w}+1\right)}\;}{2\;⎣\sqrt{\gamma_{d}^{D}-\;\sqrt{\gamma_{d}^{P}\frac{\gamma_{w}^{D}}{\gamma_{w}^{P}}}}⎦}$$

(3) $${\left(\gamma_{S}^{P}\right)}^{0.5}=\;\frac{\gamma_{w}\left(cos\theta_{w}+1\right)-2\;\sqrt{\gamma_{S}^{D}\gamma_{w}^{D}}\;}{2\;\sqrt{\gamma_{w}^{P}}}$$

where $\gamma_{S}^{D}$ and $\gamma_{S}^{P}$ are the dispersive (*D*) and polar (*P*) components of the surface free energy of the analyzed materials, respectively, γ_d_ is the surface free energy of diiodomethane (*d*); $\gamma_{d}^{P}$ and $\gamma_{d}^{D}$ are the polar and dispersive components of the diiodomethane surface free energy, respectively; $\gamma_{w}$ is the surface free energy of water (w); $\gamma_{w}^{P}$ and $\gamma_{w}^{D}$ are the polar and dispersive components of the water surface free energy; and *θ_d_* and *θ_w_* are the contact angles of diiodomethane (*d*) and water (*w*), respectively [31].

#### 2.3.7. Investigations on the Mechanical Properties of the Hydrogels

Studies on the mechanical properties of the obtained materials involved determining their tensile strength under the stress applied. Analyses were performed according to the following standards: ISO 527-2 type 5A and ISO 37 type 2. During the preparation of the hydrogel materials for mechanical tests, paddle-shaped samples were cut using a ZCP020 blanking die and placed between the jaws of a Brookfield CT3 texture analyzer. For all materials, the dependance between the tension and the deformation was determined as well as the tensile strength (*R_m_*) and the elongation as a percentage of the initial dimensions (*A*). Parameters *R_m_* and *A* were calculated using the Equations (4) and (5):

(4) $$R_{m}=\;\frac{F_{m}}{S_{0}}$$

(5) $$A=\;\frac{100*\left(l_{u}-l_{0}\right)}{l_{0}}$$

where *F_m_* is the maximum strength, *S*_0_ is the cross-sectional area of the tested sample in its initial state, *l_u_* is the measuring length after the sample’s rupture, and *l*_0_ is the measuring length of the sample in its initial state.

#### 2.3.8. Studies Aimed at Determining the Release Profile of the Active Substance from the Hydrogel Matrices

Considering the application of the developed polymers as potential drug carriers, it is essential to determine the release profile of the active substance from the interior of such materials. The curves of the release of *Aloe vera* were verified both for the hydrogels with and without albumin particles to determine the impact of this modifier, and thus the impact of the composition on the release process. Importantly, the study was performed in a neutral environment (phosphate buffer, pH = 7.4), a slightly acidic environment (acetate buffer, pH = 5.5), and an acidic environment (2% citric acid solution, pH = 2.0) to check in which conditions the release is more efficient. *Aloe vera* juice is characterized by a rich chemical composition, wherein polysaccharides were selected as substances whose amount was verified in the tested environments. For this purpose, a potassium iodide was used because this inorganic salt forms colorful complexes with polysaccharides whose presence may be subsequently verified spectrophotometrically.

In order to perform the measurements, hydrogel samples containing 10 mL of the *Aloe vera* juice (as presented in Table 1) were introduced into the flasks containing 200 mL of the tested liquids and were next placed in a laboratory shaker (Hanchen ES-60E Temperature Controlled Incubator & Shaker Scientific Incu-Shaker Shaking Incubator). The study was conducted at 36.6 °C (temperature simulating conditions occurring in the human body) and at a shaking speed of 80 rpm. At appropriate time intervals, 3 mL of each tested liquid was taken, treated with 0.125 mL of KI, and maintained for 30 min to allow time for a reaction between the potassium iodide and the polysaccharides from the released *Aloe vera* juice. After this time, a spectrophotometric analysis was performed wherein the flasks with the tested samples were supplemented with 3 mL of the appropriate liquid (citric acid solution or phosphate buffer) to maintain the same volume of the tested mixture. Spectrophotometric analysis was performed at room temperature and the maximum absorbance was observed at 310 nm.

During the spectrophotometric analysis, it was assumed that the absorbance of *Aloe vera* juice in the amount in which this modifier was introduced into the analyzed hydrogels (10 mL) was 100% (A_0_). Then, absorbances of every tested liquid were compared to the A_0_ and as a result graphs presenting the amount of the active substance released from analyzed hydrogels at appropriate time intervals were prepared.

#### 2.3.9. Studies on the Cytotoxicity of the Hydrogels via MTT Reduction Assay

An important aspect of the performed investigations was the analysis of the cytotoxicity of the obtained hydrogels. Such a study provides information on whether the applied synthesis methodology is adequate and, in turn, whether the developed materials may be considered as appropriate for biomedical uses. The analysis was performed using murine fibroblasts L929 and using MTT reduction assay, which is a standard enzymatic test used to determine the cytotoxicity of materials with medical potential. It allows to determine the amount of the living cells based on their enzymatic activity. For this purpose, the soluble 3-(4,5-dimethylthiazol-2-yl)-2,5-diphenyl tetrazolium bromide (defined as MTT reagent) was introduced into the tested environment. This salt is converted into insoluble formazan. The reaction proceeds in the presence of the enzyme—i.e., mitochondrial dehydrogenase—which is secreted by living cells. Formed formazan crystals are then dissolved in DMSO (or isopropanol) while the color intensity of this solution is evaluated spectrophotometrically and corresponds to the amount of formed formazan crystals and thus to the amount of the enzyme secreted by the tested cells (and thus corresponds to the viability of these cells). Before the investigations, the hydrogels were cut into pieces with sizes corresponding to 1/10 of the well surfaces and introduced into 5 mL of a previously prepared substrate (RPMI–1640 medium supplemented with penicillin (100 U/mL), inactivated bovine serum (10% wt.), and streptomycin (100 μg/mL) and incubated for 24 h. Then, a cell monolayer was prepared via placement of 100 μL of cell suspensions with concentrations of 2 × 10^5^ cells/mL in each well in a 96-well plate and further incubation of these cells for 24 h in standard conditions (5% CO_2_, 37 °C, >90% humidity). Next, the hydrogel samples were introduced into the wells. Finally, 20 μL of the MTT reagent was added into each well and was incubated for 4 h in standard conditions. Subsequently, a 96-well plate with tested samples was centrifuged (10 min, 1200 rpm) and the supernatant was removed. Finally, the formed formazan crystals were dissolved in 150 μL of DMSO, then in 25 μL of glycine buffer, and then incubated for 15 min at room temperature. Finally, 160 μL of the obtained solution was analyzed spectrophotometrically at a wavelength of 570 nm via the Spectramax Multi-Mode Microplate Reader (Thermo Fisher Scientific Inc., Waltham, MA, USA).

#### 2.3.10. Statistical Analysis of the Data of the Performed Experiments

The results of the performed experiments were subjected to statistical analysis. For this purpose, the statistical significance was calculated using the two-way analysis of variance (ANOVA) (alpha value = 5%). In the case of the wettability measurements, cytotoxicity evaluation, and mechanical investigations, the importance of factors such as the concentration of the albumin particles suspension and the amount of the albumin particles suspension introduced into the tested hydrogels was evaluated. In the case of the swelling studies, the importance of the swelling time was also verified. Importantly, in the case of all experiments, the measurements were performed three times and are presented as an average value and the standard deviation (SD).

## 3. Results and Discussion

### 3.1. Results of the Analysis of the Chemical Structure of the Hydrogels via FT-IR Spectroscopy

**Figure 2:**
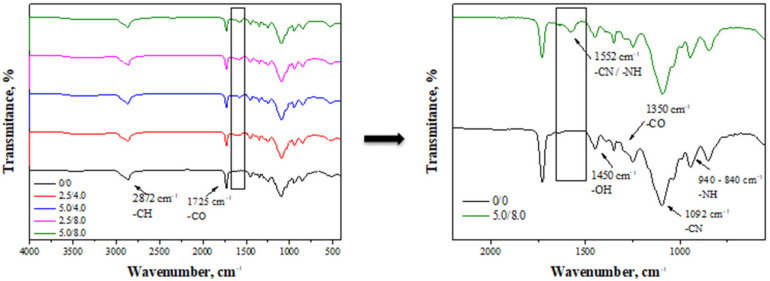
FT-IR spectra of hydrogel materials (**left**) with enlarged range of visible changes observed on the spectra of modified hydrogels (**right**).

FT-IR spectra obtained as a result of the analysis of the hydrogels are presented in Figure 2.

The absorption bands observed on the FT-IR spectra derive mainly from bonds occurring in the structure of chitosan, which is a main component of the tested hydrogel matrices [26]. For example, the band at a wavelength of approximately 2872 cm^−1^ may be assigned to the deformation vibrations of the C-H bond. Next, the band at 1450 cm^−1^ derives from the hydroxyl groups presented in the structure of chitosan. In turn, the band at 1093 cm^−1^ of a relatively high intensity compared to the that of the other bands on the spectra may be assigned to the deformation vibrations of the -CN group. Next, the bands within the range of 940–840 cm^−1^ probably correspond to the amino groups of chitosan. Analyzing the spectra, it is also possible to observe the band at 1725 cm^−1^ which likely derives from the crosslinking agent (PEGDA) present in the structure of the crosslinked hydrogels. All mentioned absorption bands may be observed on the spectra of all tested samples. Nonetheless, in the case of the hydrogels modified with albumin, an additional band at approximately 1552 cm^−1^ (Figure 2, on the right) may be identified. Such a band is characteristic of amide II bonds and corresponds to C-N stretching coupled with N-H bending vibrations. Due to the fact that such bonds are characteristic of peptide bonds occurring between amino acids in the structure of albumin, the presence of this band on the spectrum of the modified hydrogels confirms the presence of albumin in the tested materials [32].

### 3.2. Results of the Raman Spectroscopy

**Figure 3:**
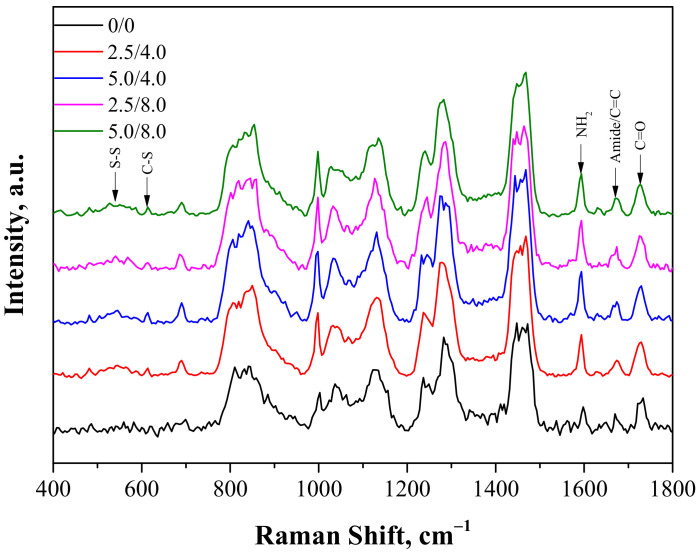
Raman spectra of hydrogels.

The next study performed involved the characterization of hydrogels obtained via Raman spectroscopy. Analysis was conducted both for the unmodified and modified polymers. The Raman spectra obtained are shown in Figure 3.

The Raman vibration spectra of the samples showed fingerprint PEGDA regions with a maximum near 1650 cm^−1^ related to the C=C bond and near 1460 cm^−1^ and 1130 cm^−1^ related to scissor and twisting CH_2_ vibrations. Next, two characteristic adsorption bands for the structure of chitosan were also observed: one with a maximum near 1590 cm^−1^ related to NH_2_ vibrations and the other with a maximum around 1670 cm^−1^ related to amide I bonds. As was reported in the case of the FT-IR analysis, the appearance of some new peaks for the hydrogels containing albumin was observed. One broad band between 500–600 cm^−1^ related to disulfide bridges S-S present in the structure of albumin appeared. Next, the second observable band with low intensity and a maximum around 610 cm^−1^ corresponding to the C-S bond, common in proteins, was also reported. Moreover, a small increase of intensity near 1670 cm^−1^ related to the presence of the amide I band was also visible. In the case of the modified hydrogels, the overlapping of bands corresponding to the C=C bond and amide bonds deriving from PEGDA and chitosan were observed.

### 3.3. Results of the Investigations on the Hydrogels’ Crystallinity via the XRD Technique

**Figure 4:**
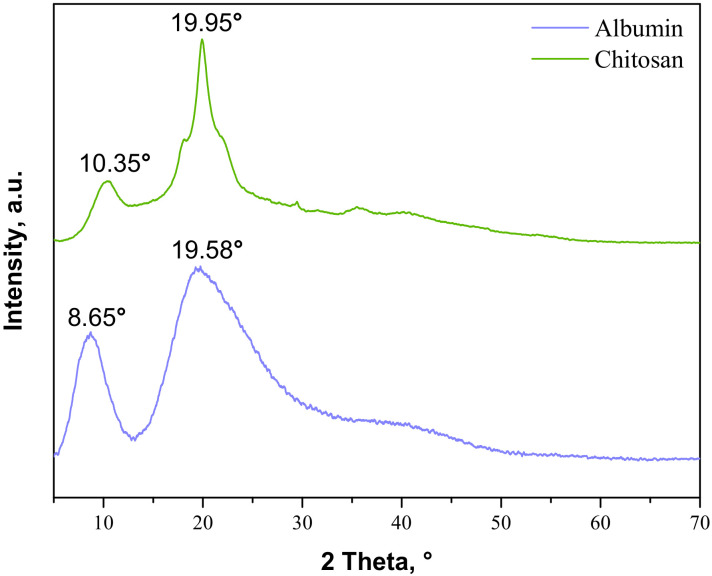
XRD patterns of commercial albumin and chitosan.

The next part of the study was focused on determining the changes in the crystallinity of the samples during the tensile load. Firstly, the analysis was performed for commercial materials, i.e., chitosan and albumin; the obtained XRD patterns are presented in Figure 4.

**Figure 5:**
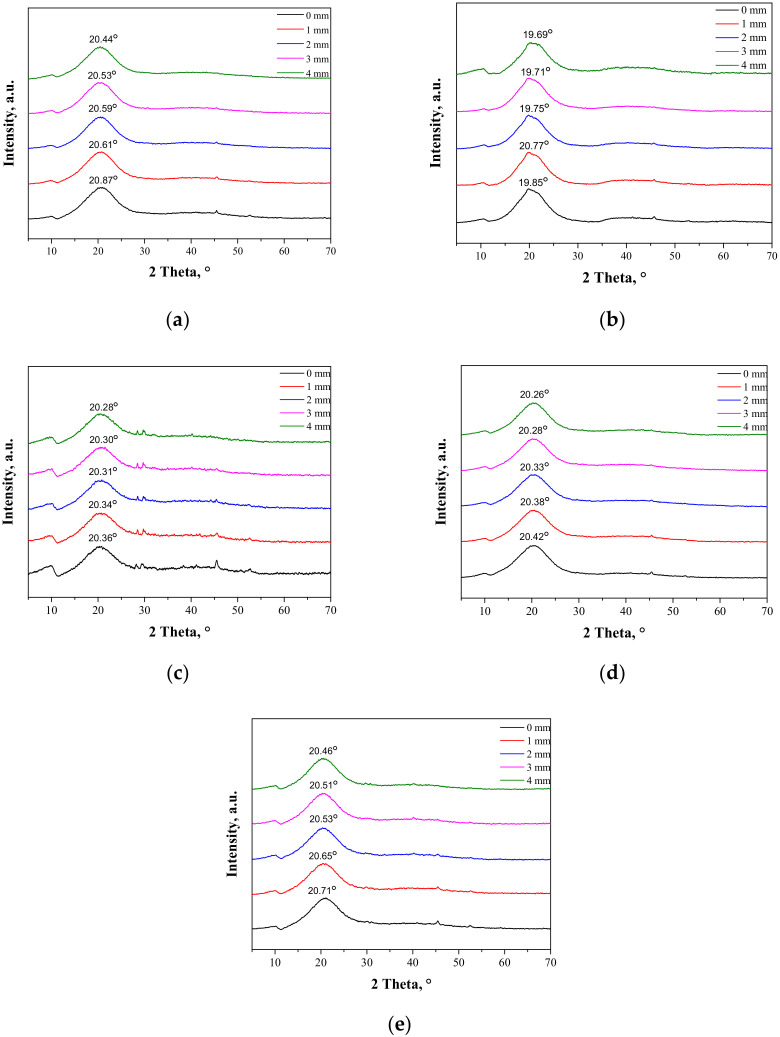
XRD patterns of sample 0/0 (**a**); sample 2.5/4.0 (**b**); sample 5.0/4.0 (**c**); sample 2.5/8.0 (**d**); and sample 5.0/8.0 (**e**) subjected to the tensile load.

As may be observed, the chitosan showed characteristic diffraction peaks around 2θ: 10.35°, 19.95°, and 21.90° corresponding to the planes (020), (110), and (120), respectively. In the albumin diffractogram, two broad diffraction peaks around 2θ: 8.65° and 19.58° may be observed. They are related to the occurrence of structure ordering in the sample, i.e., intermolecular H-bonding interactions between protein chains. These XRD patterns are presented as a reference in Figure 5. The XRD patterns of the prepared hydrogels during the tensile load are presented.

**Figure 6:**
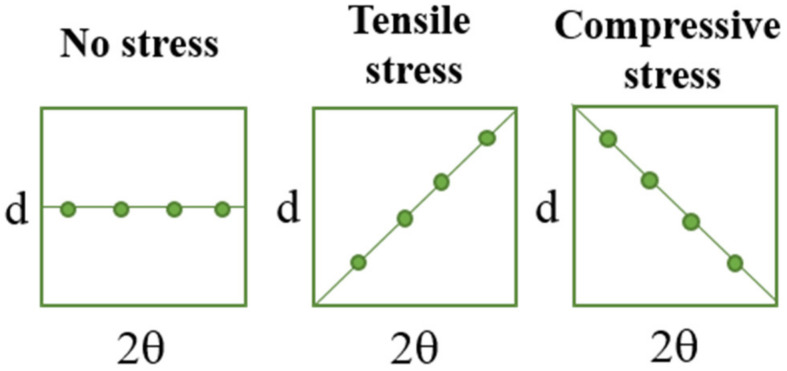
The simplified schemes presenting the changes of d and 2θ parameters under the tensile load.

In Figure 6, the schemes presenting the changes of d and 2θ parameters under stress are presented to visualize better the methodology applied.

During the extension of the samples, no changes in the form of structure ordering were observed. Only slight shifts of the signals were visible, which was related to the change of the interplanar distances (d-spacing). This value, according to Bragg’s equation, has an influence on the positions of the signals on the diffractometer. When compressive stresses are visible, the d value decreases, while tensile stress causes an increase in the d value (Figure 5).

The maximal force detected for samples B-E was within the range of 7–9 N, while only sample A needed the application of a higher force, i.e., 12.4 N. The break was only detected for sample A at an extension of approximately 3.8 mm, which corresponded to strain around 26%. Other samples did not break at 27.5% strain, which proves the positive effect of albumin addition on the plastic behavior of the hydrogels.

### 3.4. Morphological Analysis of the Hydrogels via SEM Technique

**Figure 7:**
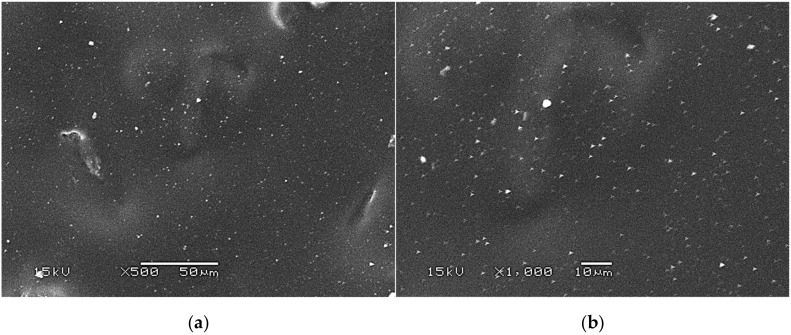
SEM images of unmodified hydrogels at magnification ×500 (**a**) and ×1000 (**b**).

**Figure 8:**
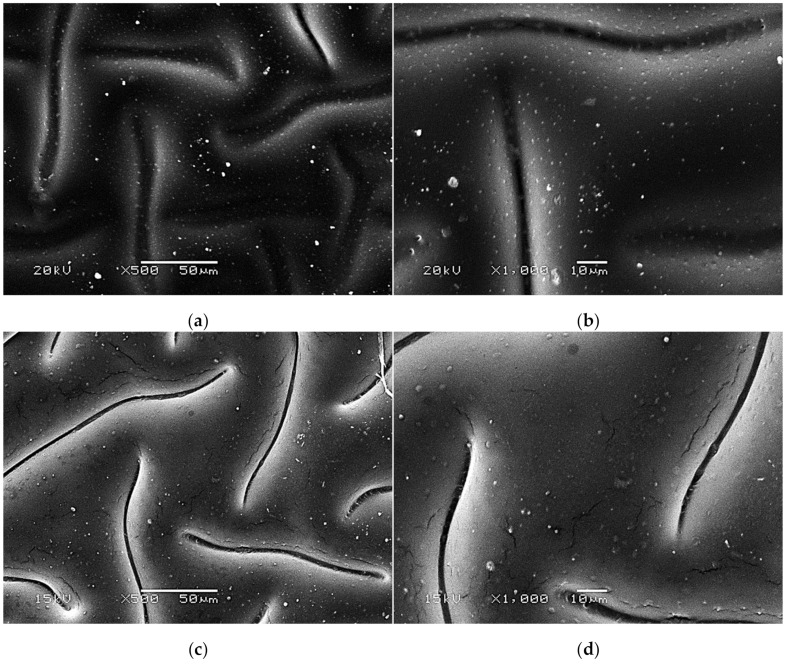
SEM images of modified hydrogels at various magnifications: sample 2.5/4.0 (×500) (**a**); sample 2.5/4.0 (×1000) (**b**); sample 5.0/4.0 (×500) (**c**); sample 5.0/4.0 (×1000) (**d**).

**Figure 9:**
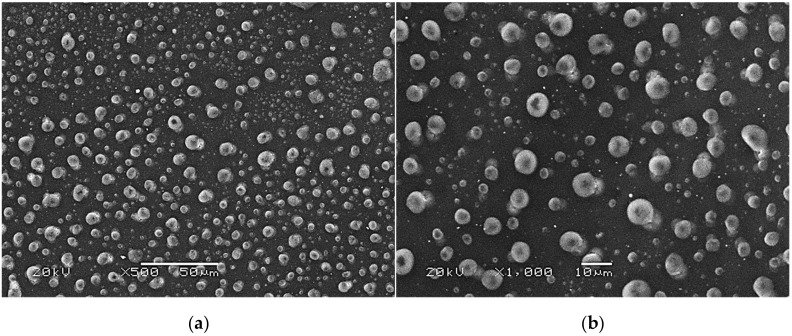

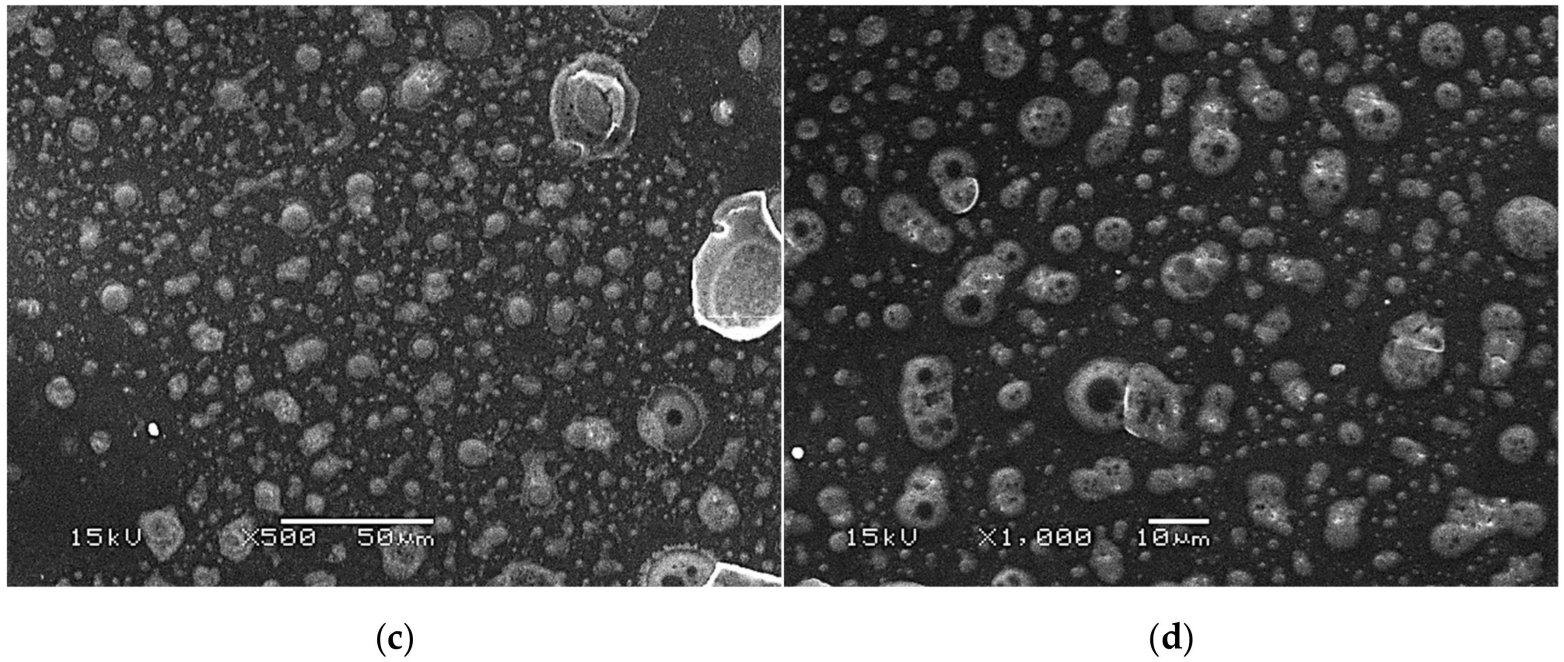
SEM images of modified hydrogels at various magnifications: sample 2.5/8.0 (×500) (**a**); sample 2.5/8.0 (×1000) (**b**); sample 5.0/8.0 (×500) (**c**); sample 5.0/8.0 (×1000) (**d**).

SEM images presenting the surface morphology of the hydrogels are presented below in Figure 7, Figure 8 and Figure 9.

The SEM technique was performed to characterize the morphology of both the unmodified hydrogels and those modified with albumin particles. As may be observed in the obtained images, the unmodified hydrogel sample showed a smooth and homogeneous surface. Different observations may be reported based on the SEM images of the modified polymers; thus, it may be concluded that the additive introduced into the hydrogels affected their surface morphology. In the images showing the surface morphology of the hydrogels modified with albumin with a higher concentration (samples 2.5/8.0 and 5.0/8.0), numerous circular shapes are visible, which probably corresponds to the albumin particles. Such elements were not noticeable in the case of hydrogels containing albumin with a lower concentration (samples 2.5/4.0 and 5.0/4.0). It is assumed that the use of albumin with various concentrations affected the salt-induced precipitation process. As a result, albumin spheres with various sizes depending on the concentration were formed. The use of albumin with a higher concentration may lead to the preparation of larger sized albumin spheres, while in the case of the use of this protein with a lower concentration, smaller spheres were obtained, even having nanometric sizes that were confirmed in our previous publication [28]. This is why these small albumin particles (e.g., nanometric ones) are difficult to observe on the surface of the modified hydrogels in contrast to the larger particles clearly visible in the SEM images. Nonetheless, the presence of these smaller particles was verified via spectroscopic techniques (FT-IR and Raman).

### 3.5. Investigations on the Swelling Properties of the Hydrogels

**Figure 10:**
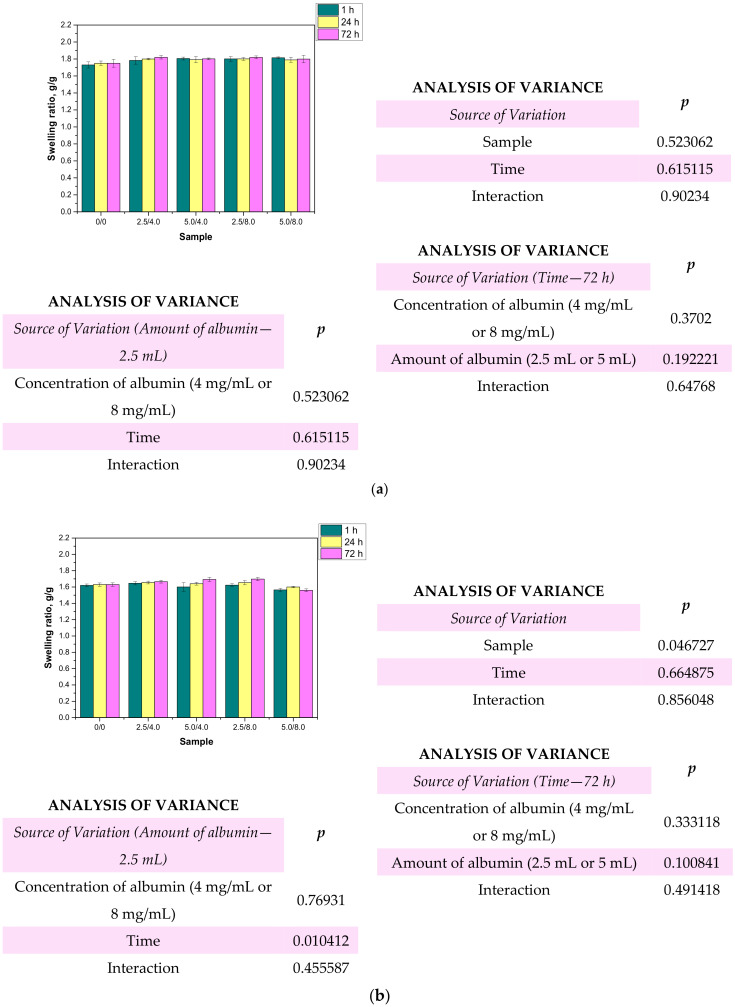

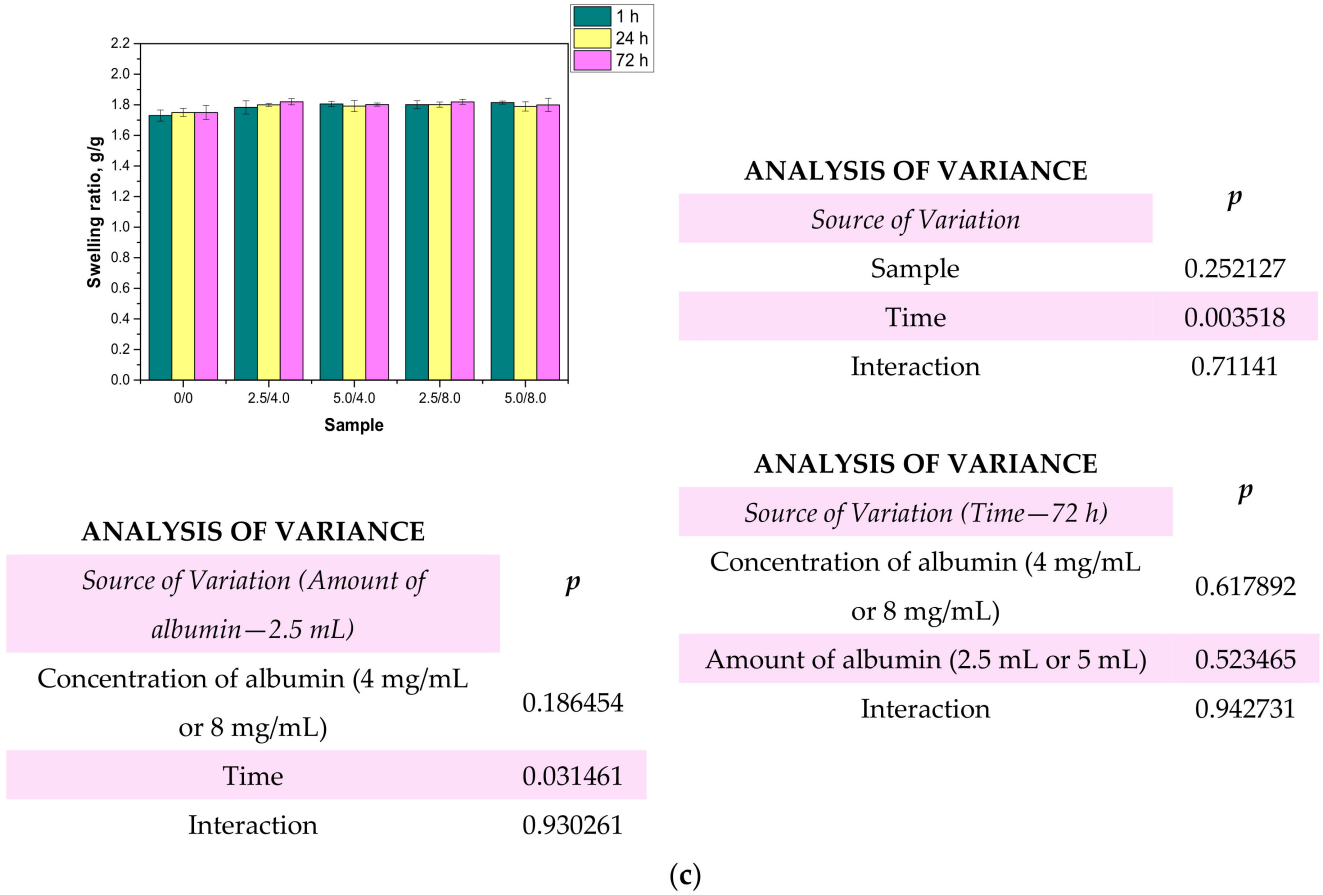
Results of swelling studies performed in distilled water (**a**), SBF (**b**), and Ringer liquid (**c**) (n—number of repetitions, n = 3; *p* indicates the statistical significance calculated using the two-way analysis of variance (ANOVA).

One of the most characteristic features of hydrogel materials is their ability to absorb large quantities of liquids. Determining the swelling properties of the hydrogels was significant in view of the fact that the developed polymers were being considered for biomedical applications, for example, as wound dressing materials. It is assumed that dressings should absorb wound exudates that may accelerate the wound healing process. The sorption ability of the materials determined by swelling ratios α is presented below in Figure 10. The study was performed both for the unmodified and modified hydrogels.

Based on the obtained results, it may be stated that all the tested hydrogels showed swelling capability, so they may absorb wound exudates. The highest sorption ability was observed after 1 h of the study. After the next 24 and 72 h, the swelling ratios calculated for all samples slightly changed compared to their values determined after 1 h. The highest swelling ratios were calculated in the case of the studies performed in distilled water. For example, the value of α for hydrogel sample 0/0 swelling in this liquid for 1 h was 1.73 g/g. Slightly lower values of the swelling ratios were determined for samples tested in SBF and Ringer liquid, i.e., α = 1.62 g/g for SBF and α = 1.63 g/g for Ringer liquid, respectively. These differences may be caused by the various compositions of liquids in which the study was performed. Liquids such as SBF or Ringer liquid contain various ions which may contribute to the formation of additional crosslinks in the polymer matrix. This, in turn, leads to the increase in the crosslinking degree of the hydrogel and finally to the decrease in the free spaces between polymer chains which are available for absorbed liquid. Therefore, the highest swelling capacity was determined in distilled water where any ions are present. Additionally, an essential aspect was determining the impact of the modifying agent on the swelling properties of the analyzed hydrogels. Comparing the results obtained for the unmodified hydrogels and those containing albumin, it was proved that the presence of albumin in hydrogel matrices did not reduce the swelling capacity of the modified materials. The differences between the values of the swelling ratios calculated for the modified hydrogels and the hydrogels without albumin were slight. Furthermore, the swelling ratios of the modified polymers were slightly higher, so it was proved that such a modification resulted in better sorption properties, which is favorable in the viewpoint of their potential application.

### 3.6. Assessment of the Wettability of the Hydrogels

In this analysis, two liquids, i.e., bipolar water and polar diiodomethane, were used to characterize the surface free energy of the examined hydrogels. For this purpose, the Owens–Wendt method was used, which assumes that interactions between molecules of two substances present in their surface layer are equal to the geometric average of intermolecular interactions within each substance. A detailed introduction to this method was presented by Rudawska et al. [31]. According to the assumptions of this method, the polar and dispersive components of the tested materials can be calculated using the Equations (2) and (3) [31].

**Figure 11:**
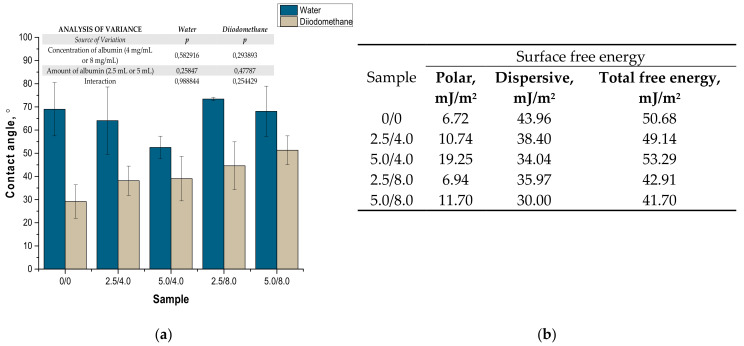
Values of contact angles (**a**) (n—number of repetitions, n = 3; *p* indicates the statistical significance calculated using the two-way analysis of variance (ANOVA)) and surface free energies determined for tested hydrogels (**b**).

The values of the contact angles determined as well as surface free energies calculated based on the contact angles are presented in Figure 11. The presented results constitute an average value of three measurements. The presented values of the contact angles constitute an average value of three measurements.

**Figure 12:**
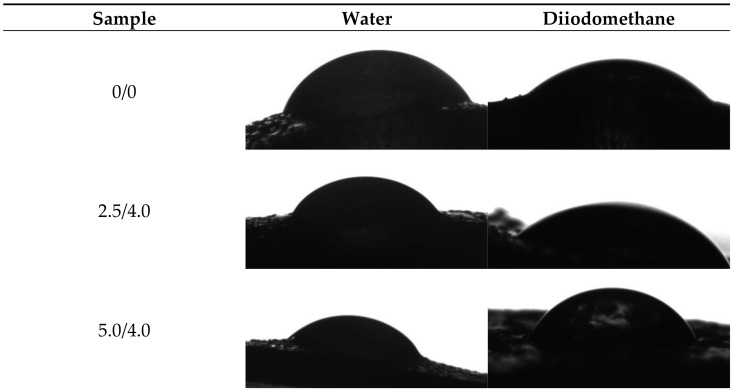

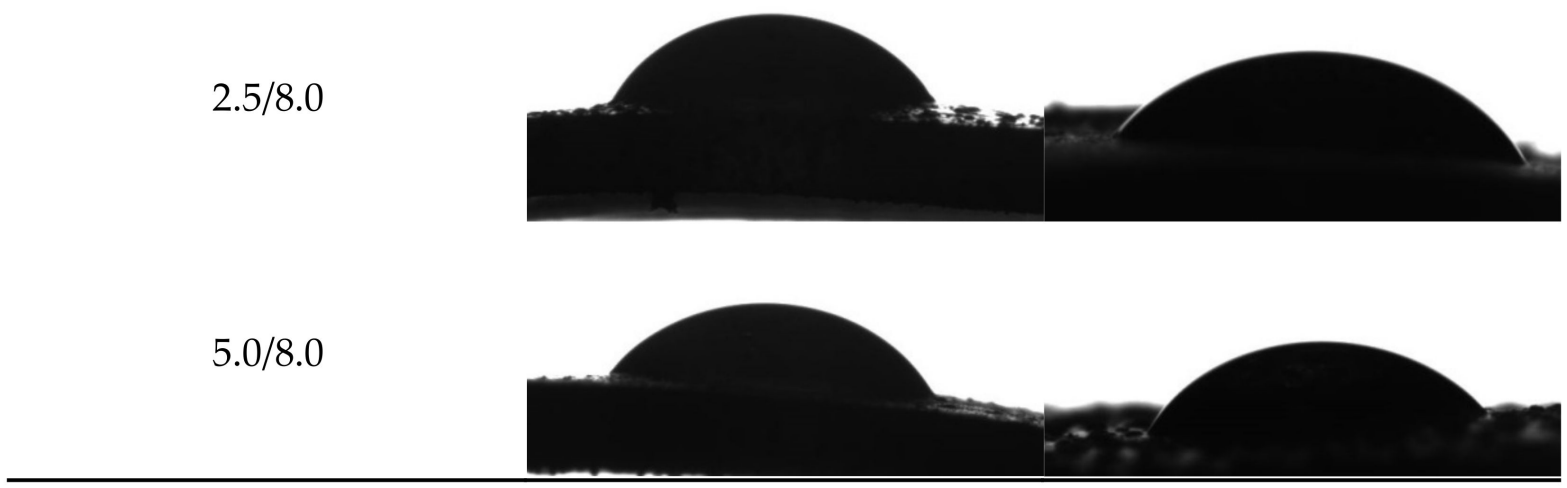
Images showing the wettability of hydrogels towards selected liquids.

Example images showing the first contact of the drop liquid with the surface of the tested materials are presented in Figure 12.

Due to the potential biomedical application of the developed hydrogels, including as innovative wound dressings, it is important to determine the behavior of such materials in contact with water. One of the main tasks of hydrogel dressings is to provide a moist environment for the wound. On the other hand, a good surface wettability indicates the materials’ hydrophilicity and is strictly related to the cell proliferation. It is assumed that more intensive growth and development of epithelial cells takes place in the case of materials whose surfaces are well wetted with water [33]. For example, the proliferation and spread of epithelial cells on hydrophilic and hydrophobic surfaces were presented in studies performed by An et al. They proved that a better growth of fibroblasts occurs on hydrophilic surfaces [34].

The tested surface is defined as hydrophilic when the contact angle of water in contact with such a surface is lower than 90° [35]. The contact angles measured for all tested hydrogels were ˂90°; therefore, it may be concluded that the hydrogels obtained were materials well wetted by water. Furthermore, the impact of the modifying agent of the hydrogel matrix may be noticed. The lowest contact angle—so the better wetting properties—was observed for the hydrogel sample modified with 5.0 mL of albumin particles with a concentration of 4 mg/mL. In the case of samples containing albumin with a concentration of 8 mg/mL, the contact angles were higher while the surface free energies were lower than in the case of the materials modified with an albumin suspension with a lower concentration. It is assumed that the use of albumin particles with various concentrations in the salt-induced precipitation affected their size. In the case of higher concentrations, albumin particles with larger diameters were expected, which limits the spreading of water on the surface of the tested material. In the case of albumin particles with lower diameters which may distribute homogenously in the tested material, an increase in the wettability of the material was observed. This, in turn, is consistent with the conclusions drawn based on the SEM imaging.

### 3.7. Results of the Evaluation of the Mechanical Properties of the Hydrogels

**Figure 13:**
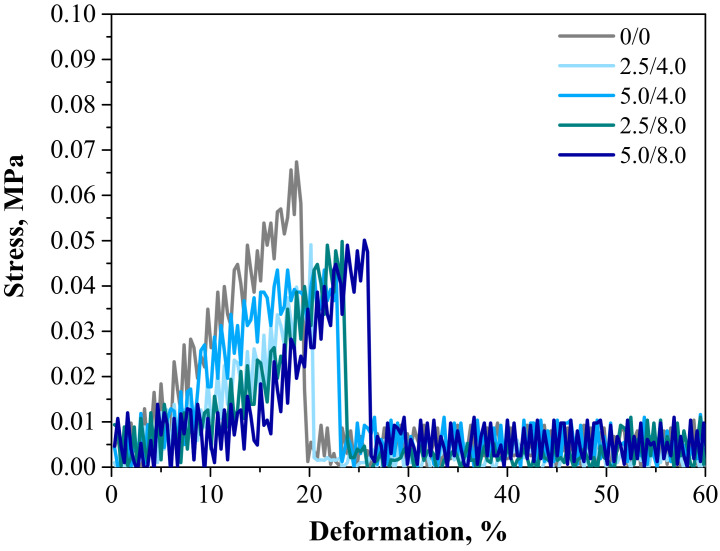
Stress–strain characteristics of hydrogels.

**Figure 14:**
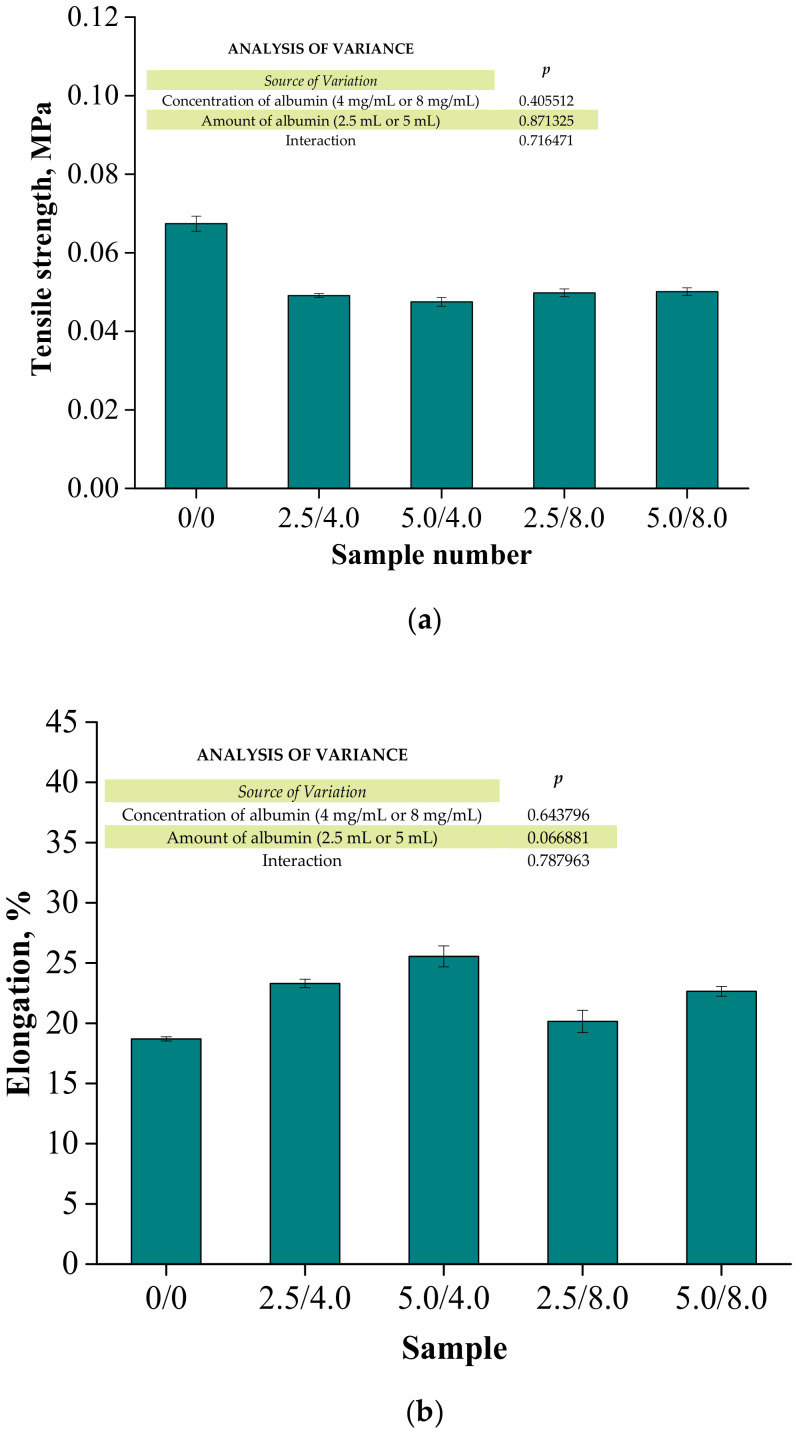
Results of the mechanical evaluation of tested materials: tensile strength (**a**) and percentage elongation (**b**) (n—number of repetitions, n = 3; *p* indicates the statistical significance calculated using the two-way analysis of variance (ANOVA)).

Below are the results of the mechanical investigations of both unmodified hydrogels and those containing albumin particles. Figure 13 shows a stress-deformation curve, Figure 14a presents tensile strengths of the hydrogels, and Figure 14b reflects the percentage of elongation of the tested polymers.

Investigations were performed to determine the impact of the introduced modifying agent on the mechanical properties of the modified hydrogels. This is the reason why both unmodified polymers and polymers modified with albumin particles were subjected to these studies. The highest tensile strength was reported for the unmodified sample (R_m_ = 0.0674 MPa). Nonetheless, the lowest elongation (A = 18.70%) corresponding to the lowest elasticity was also measured for this sample. Next, it was observed that the modification of hydrogels with albumin particles resulted in a decrease in their tensile strength and a simultaneous increase in their elasticity. This indicates that the application of lower tension—thus, a lower tensile strength—is sufficient for stretching of the modified hydrogels compared to the unmodified materials. Importantly, analogous conclusions were drawn during the investigations on the crystallinity of the hydrogels subjected to the tension load described in Section 3.3. Here, the stretching of the unmodified polymers required the application of an adequately higher tension load compared to the stretching of samples containing albumin (F = 12.4 N for unmodified samples, F = 7–9 N for modified samples, respectively). In general, hydrogel polymers incorporated with albumin spheres showed higher elasticity than unmodified materials, which is also in line with the results presented in Section 3.3. Moreover, based on the obtained results, it was reported that albumin spheres prepared using lower concentrations of albumin solution were characterized by the highest elasticity with relatively high tensile strength (Rm = 0.0501 MPa, A = 25.5% for sample 5.0/8.0). Considering the application of hydrogels as dressing materials, the developed materials need to be characterized by both the appropriate tensile strength and elasticity. Furthermore, the ability to control these properties by selecting the appropriate amounts of introduced albumin spheres is an advantage of such materials that increases the area of their potential applications.

### 3.8. Results of the Investigations on the Release of the Active Substance from the Developed Materials

**Figure 15:**
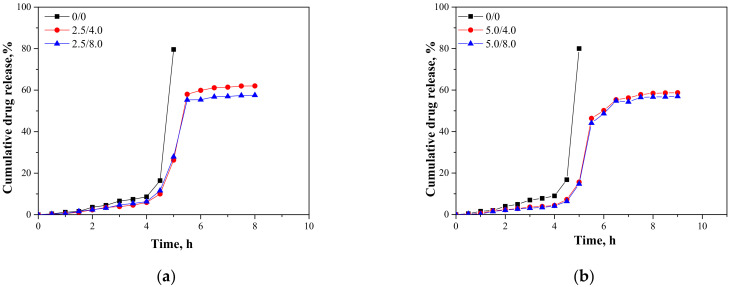

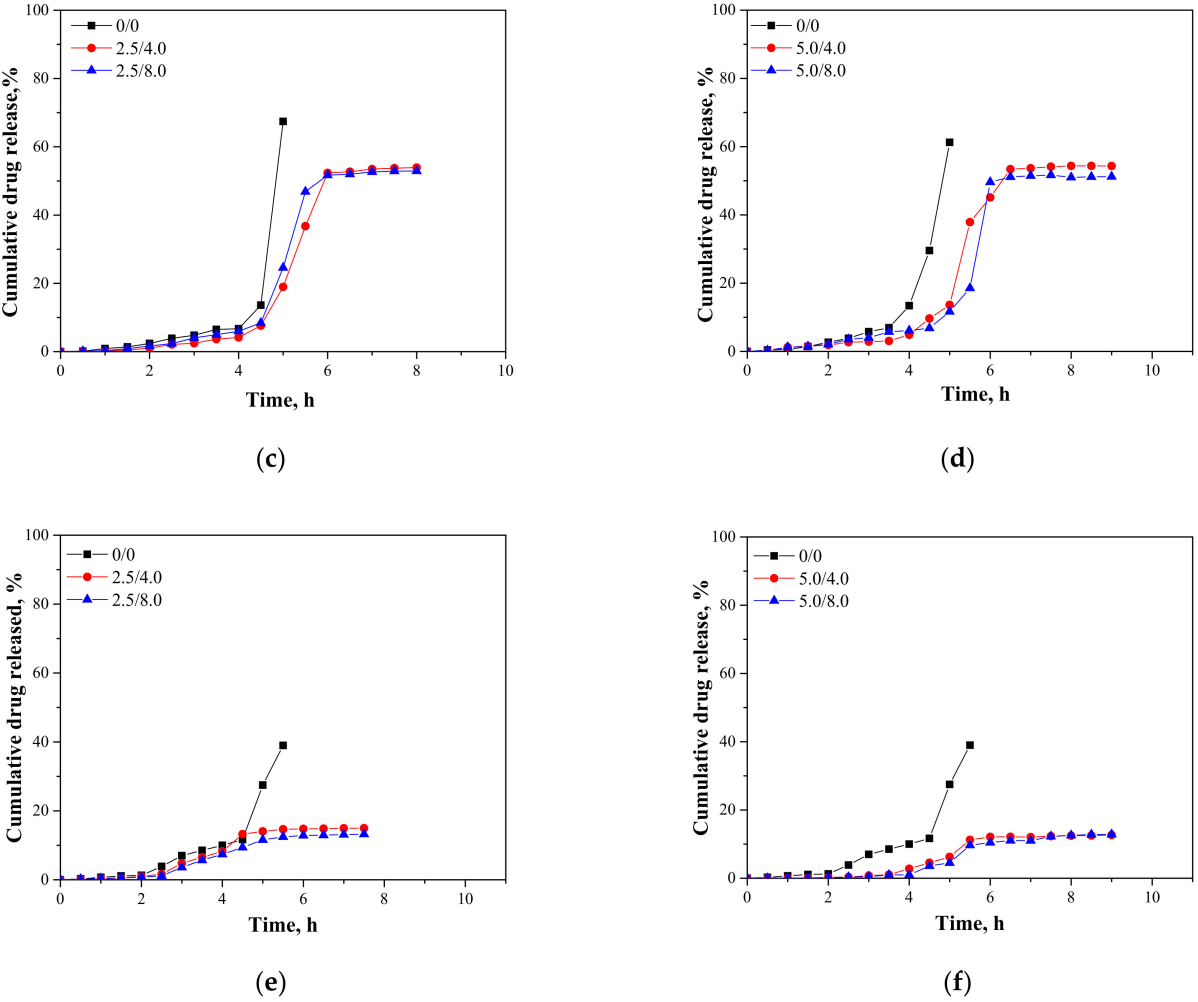
Release profiles of active substance from hydrogels in environment at pH = 2.0—samples containing 2.5 mL (**a**) and 5.0 mL (**b**) of albumin spheres; pH = 5.5—samples containing 2.5 mL (**c**) and 5.0 mL (**d**) of albumin spheres; and pH = 7.4—samples containing 2.5 mL (**e**) and 5.0 mL (**f**) of albumin spheres.

In Figure 15, results presenting the release profiles of the active substance from the analyzed materials (as cumulative drug release)—both from hydrogels containing 2.5 mL and 5.0 mL of albumin spheres suspension—are shown. The studies were performed for 10 h in environments at pH = 2.0, 5.5, and 7.4, wherein the results were presented until the release of the active substance stopped. 

An analysis was performed to determine the amount of the released active substance in selected time intervals. The study was performed both for unmodified hydrogels (without albumin) as well as for polymers containing 2.5 mL and 5.0 mL of albumin spheres prepared using albumin solution with concentrations of 4.0 mg/mL and 8.0 mg/mL, respectively. It was reported that in both all the tested environments significant differences between the release of the active substance from the unmodified hydrogel and hydrogels containing albumin spheres were observed. In the case of the unmodified matrix, a determined release profile indicated the rapid release of the active substance after 5 h of the study. Then, a rapid release of the highest amount of the active substance was observed, after which further release was not reported. The release of *Aloe vera* from samples modified with albumin spheres was also the most effective after approximately 5 h of the research. Nonetheless, despite the fact that a lower amount of the active substance was released from the modified polymers, such a release profile is more favorable in the viewpoint of the potential application of the developed materials. In this case, an intense release of the active substance (i.e., 60% in an acidic environment) was observed for a few hours; thus, it may be defined as an extended-release profile. Therefore, considering the potential applications of the developed hydrogels, e.g., as dressing materials, the ability to modify such a material to reach an extended-release profile is promising and meaningful because in some cases an extended drug release is more favorable. Such a process is, in turn, caused by many interactions occurring between a chitosan-based matrix and an introduced albumin. It is assumed that the formation of protein-polysaccharide complexes is a result of intermolecular electrostatic interactions, hydrogen bonds, and hydrophobic interactions [36]. Positively charged chitosan interacts with negatively charged albumin. Combinations of these biopolymers and the interactions between them were also discussed by Niaz et al. and Karimi et al. [37,38]. In the case of the modified materials, interactions between a chitosan matrix and protein spheres resulted in the formation of a hydrogel matrix with a more compact structure compared to the structure of the unmodified material. Thus, the release of *Aloe vera* is more difficult and limited than for the unmodified polymer, and finally an extended release is observed. The extended-release profile compared to the unmodified hydrogel sample was observed both for samples modified with 2.5 mL and 5.0 mL of the albumin particles suspension. In the case of the application of a larger amount of the modifier, it was observed that the release profile of *Aloe vera* juice was further extended. The increase in the amount of albumin particles in the hydrogel matrix probably resulted in the occurrence of more intense interactions between the functional groups of this protein and chitosan, which, in turn, resulted in the formation of a more compact hydrogel structure. The highest amount of the active substance released from sample 2.5/4.0 at pH = 5.5 was 53.91%, while for sample 5.0/4.0 it was 51.21%. Nonetheless, analyzing the release process from the same samples in t = 9 h. it may be noticed that in the case of sample 5.0/4.0 the release of *Aloe vera* in this period of time was maintained at 51.21%, while for samples containing lower amounts of albumin spheres, release after 8 h was not noticed. Thus, it may be concluded that the introduction of a higher amount of the modifier resulted in the release of a slightly lower amount of *Aloe vera* juice and simultaneously a more extended-release profile.

Considering the various environments of the research (i.e., acidic and neutral), a significantly more effective release was observed in the acidic environment. Here, -NH_2_ groups from the polymer chains of chitosan were protonated, which led to the formation of -NH_3_^+^ ions. These cations, in turn, repelled each other, and as a result a loosening of the polymer structure may be observed increasing at the same time as free spaces in the polymer network. This led to the easier release of *Aloe vera* from such a network to the tested medium. In a slightly acidic environment (pH = 5.5), such interactions also took place but to a much lesser extent; thus, cumulative drug release was less intense in this environment than in the case of the release observed for pH = 2.0. In turn, in a neutral environment, such a loosening was not observed; thus, the release of *Aloe vera* was less effective. Considering the possible applications of the developed materials, e.g., as carriers of cytostatic drugs, the more effective release in an acidic environment is highly desirable. Albumin shows a tendency to accumulate near neoplastic cells; thus, this protein may be successfully applied as a carrier for chemotherapeutics. Importantly, the pH of the environment within cancer cells is lowered; thus, the results of the performed investigations, which indicate a more effective release in exactly such conditions, are promising and indicate the application potential of the developed materials for this purpose.

### 3.9. Results of the Evaluation of the Cytotoxicity of the Developed Materials

**Figure 16:**
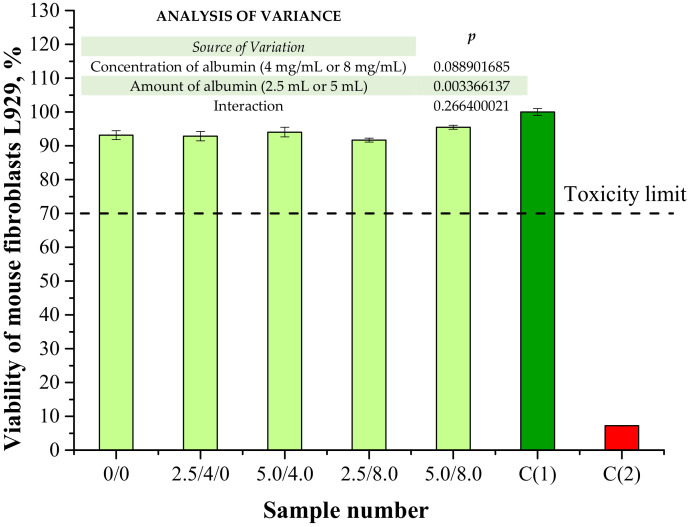
Results of cytotoxicity evaluation via MTT reduction assay (n—number of repetitions, n = 3; *p* indicates the statistical significance calculated using the two-way analysis of variance (ANOVA)).

In the viewpoint of the potential application of the obtained hydrogels for biomedical purposes (i.e., as dressing materials), one of the most important investigations was analysis of their cytotoxicity towards the selected cell lines. The results of a cytotoxicity assessment performed using MTT reduction assay are presented below in Figure 16. The study was also conducted for a well-known non-cytotoxic biomaterial (C(1)) and a phenol solution showing cytotoxicity (C(2)) for reference purposes.

According to ISO recommendations for materials designed for biomedical purposes, when the viability of the tested cell lines incubated with the analyzed material is >70% for 24 h, then such a sample is defined as non-cytotoxic [39]. In the case of both unmodified hydrogels and polymers containing albumin spheres, the viability of mouse fibroblasts L929 was approximately 91–95%. Therefore, it was proved that the developed hydrogel materials did not exhibit cytotoxic properties. Moreover, results of the performed investigations indicated that the albumin spheres had no significant impact on this property. The lack of a negative impact from the introduced protein spheres may indicate that the method of preparation of the albumin spheres as well as their purification supported by their washing with distilled water were appropriate.

## 4. Conclusions

- Both Raman and FT-IR spectroscopic analyses confirmed the presence of albumin in hydrogel matrices due to the presence of many bands corresponding to the protein structures on the obtained spectra.
- All hydrogels showed good swelling properties; the highest swelling was observed in distilled water. This is related to the fact that in the other tested liquids—SBF and Ringer liquid—mono- and divalent ions occur which may contribute to the formation of additional crosslinks between polymer chains which, in turn, leads to the reduction of the swelling capacity of hydrogels.
- The introduction of albumin into the hydrogels increased their sorption properties by approximately 10%.
- The modification of hydrogels with albumin particles also resulted in their increased resistance to the tensile load, i.e., approximately 30% more force was needed to break the hydrogels containing this protein. Analogous results were also obtained during an analysis of the crystallinity of the hydrogels under the tensile load via the XRD technique and mechanical tests.
- The contact angles of all the tested hydrogels were lower than 90°; thus, the hydrophilicity of the hydrogels was proved.
- The introduction of albumin spheres into the polymer matrices resulted in a preparation of drug carriers with an extended drug release profile.
- The developed hydrogels did not show cytotoxicity towards murine fibroblasts L929. Any negative impact of albumin spheres on this property was not observed. In vitro biological tests showed that the viability of the tested cells was within the range of 91—95%, which allows for the conclusion that there are no contraindications for performing more advanced biological experiments.
- Here, a main focus was to verify whether the developed hydrogel materials show an application potential for biomedical purposes. It was proved via MTT reduction assay that these polymers exhibited biological compatibility. This, in combination with their swelling properties, hydrophilicity, mechanical properties, and the ability to release an active substance from their interior makes them promising for biomedical applications and allows for the design of more advanced investigations aimed at verifying their utility, e.g., as wound dressings functionalized for delivery of specific drugs such as cytostatics and at the same time for the treatment of burn wounds. For this purpose, significant attention will be paid to the cytotoxic evaluation towards such cell lines as cancer cells or specific skin cells. The developed materials seem to be promising for such applications due to the soothing effect of *Aloe vera* juice, which is favorable in treating burns; the ability to perform drug release from hydrogel matrices; and the potential of these materials for wound exudate sorption. The affinity of albumin for neoplastic cells constitutes an additional important advantage when considering albumin-based particles as carriers of cytostatics.

## Figures and Tables

**Table 1:** Compositions of hydrogel polymers.

Base Solution, mL	Photoinitiator *, mL	Crosslinking Agent **, mL	*Aloe vera*, mL	Albumin 4.0 mg/mL Solution, mL	Albumin 8.0 mg/mL Solution, mL	Sample Name
50	0.5	12	10	-	-	0/0
				2.5	-	2.5/4.0
				5.0	-	5.0/4.0
				-	2.5	2.5/8.0
				-	5.0	5.0/8.0

* 2-hydroxy-2-methylpropiophenone, Darocur 1173. ** diacrylate poly(ethylene glycol).

**Table 2:** Concentrations of ions included in SBF solution [29].

Ion	Concentration, mmol/L	Ion	Concentration, mmol/L
Na^+^	142.0	Cl^−^	103.0
K^+^	5.0	HPO_4_^2−^	27.0
Mg^2+^	1.5	HCO_3_^−^	1.0
Ca^2+^	2.5	SO_4_^2−^	0.5

## Data Availability

The data presented in this study are available on request from the corresponding authors.
